# TechnoBrainBodies-in-Cultures: An Intersectional Case

**DOI:** 10.3389/fsoc.2021.651486

**Published:** 2021-04-27

**Authors:** Sigrid Schmitz

**Affiliations:** Gender Studies in Cologne, University of Cologne, Cologne, Germany

**Keywords:** intersectionality, neuroscience, neuro-technologies, transhumanism, neuro-governmentality, posthuman*ities*

## Abstract

The cyborgization of brainbodies with computer hardware and software today ranges in scope from the realization of Brain–Computer Interfaces (BCIs) to visions of mind upload to silicon, the latter being targeted toward a transhuman future. Refining posthumanist concepts to formulate a posthuman*ities* perspective, and contrasting those approaches with transhumanist trajectories, I explore the intersectional dimension of realizations and visions of neuro-technological developments, which I name TechnoBrainBodies-in-Cultures. In an intersectional analysis, I investigate the embedding and legitimation of transhumanist visions brought about by neuroscientific research and neuro-technological development based on a concept of modern neurobiological determinism. The conjoined trajectories of BCI research and development and transhumanist visions perpetuate the inscription of intersectional norms, with the concomitant danger of producing discriminatory effects. This culminates in normative capacity being seen as a conflation of the abled, successful, white masculinized techno-brain with competition. My deeper analysis, however, also enables displacements within recent BCI research and development to be characterized: from ‘‘thought-translation” to affective conditioning and from controllability to obstinacy within the BCI, going so far as to open the closed loop. These realizations challenge notions about the BCI's actor status and agency and foster questions about shifts in the corresponding subject–object relations. Based on these analyses, I look at the effects of neuro-technological and transhumanist governmentality on the question of whose lives are to be improved and whose lives should be excluded from these developments. Within the framework of political feminist materialisms, I combine the concept of posthuman*ities* with my concept of TechnoBrainBodies-in-Cultures to envision and discuss a material-discursive strategy, encompassing dimensions of affect, sociality, resistance, compassion, cultural diversity, ethnic diversity, multiple sexes/sexualities, aging, dis/abilities—in short, all of this “intersectional stuff”—as well as obstinate techno-brain agencies and contumacies foreseen in these cyborgian futures.

## Introduction

In a recent documentary entitled “Myth of the Artificial Brain” (Denjean, 2017)^1^, the French/German Television *ARTE France* channel presented an up-to-date account of the state of neuroscientific research and neuro-technological development, *as well as* outlining current visions of transhumanism. Human enhancement with the help of the latest scientific and technological advancements shall render more-than-human capabilities and intelligence possible, culminating in the possibility of mind upload to silicon. This is a new scenario. There are numerous popular science documentaries available covering current findings in neuroscience, including the development of Brain–Computer and Brain–Machine Interfaces (BCI/BMI) for improved treatment of patients suffering from communicative or motor impairment. There are also reams of fictional stories and films about artificial humanoids or humanoid robots. Furthermore, the transhumanist movement has disseminated its objectives (*The Transhumanist Declaration*, Various, 2013) worldwide via its internet appearances, e.g., the transhumanist party (www.transhumanist-party.org), Humanity+ (humanityplus.org), Extropianism (http://www.extropy.org/, an institute founded by Max More and Natasha Vita-More), Democratic Transhumanism (Hughes, 2004), or Singularity (Kurzweil, 2005). However, although transhumanists refer back to neuroscience and neuro-technologies, for decades, they have mostly been regarded as crackpots by established members of the neuroscientific research and neuro-technological development fields. Nevertheless, over the last few years, a new reciprocal connectivity has emerged: protagonists from both sides—the neurosciences/neuro-technologies and transhumanism—mutually refer to each other's findings, developments, and visions with a positive couleur, in particular just those interconnections popularized by the ARTE documentary. Neuroscientists predict that it will become possible to measure all functions of the brain and to explain human behavior and thinking as a whole in the near future. BCI developers connect brains with computer hardware and software for “thought” translation. A group of (neuro-)clinicians, (neuro-)engineers, computer experts, and transhumanists recently published a prognosticated Human Brain/Cloud Interface in *Frontiers in Neuroscience*, which would allow a person to get direct access to “virtually any facet of cumulative human knowledge” (Martins et al., 2019). Researchers of cryonics prophesy cryopreservation as a promising way of preserving enough brain information to permit future revival of cryopreserved persons and enable their human mind to be uploaded to silicon. In the ARTE documentary, we learn about Ken Hayworth, researcher at the Howard Hughes Medical Institute's Janelia Farm Research Campus in Ashburn, Virginia, a leading research institution in the field of connectomics^2^, who has founded the Brain Preservation Foundation^3^ with the aim of having his (sic!) brain cryopreserved after his death. After 100 years, it should be defrosted again and uploaded to silicon—as a “mind up,” so he says! Moreover, the Brain Preservation Foundation awarded a prize to a laboratory for its first cryopreservation and later defrosting of a rabbit. These examples give an impression, I would suggest, of how science–transhumanist exchange is becoming intelligible in the sense of a discursive norm (Butler, 1990).

Nothing new: the ARTE documentary presented a significant number of *white*, successful, middle-aged men, who expounded on their research and visions, from visualizing the brain's connectome to the development of neuro-technologies, along with their visions of mind upload into supercomputers or robotic counterparts. No women appeared in the documentary within this group of leading representatives of the field, apart from two staff members and a female technician, who were shown conducting some experiments. Nor do any Non-whites appear, except for Hiroshi Ishiguru, who has developed the android “Geminoid” as his robotic twin. In actual fact, that is not quite true. Transwoman Martine Rothblatt is presented as one of the richest women in the world, complete with BINA48 (Breakthrough Intelligence via Neural Architecture 48^*^), a robotic head-like chatbot in which she has stored all memories of her black female partner Bina. An admittedly non-systematic search through my literature sample in the field of BCI/BMI developments revealed three women among 32 first authors in empirical studies. In contrast, in May 2020, the international NeuroGenderings expert network^4^ embraced 88 members, 6 of them men—as far as I could assign their gender. The network connects scholars from a broad range of brain research disciplines, including neuroscience, neuropsychology, cognitive neuroscience, and epidemiology, with scholars from gender and queer studies, feminist science studies, and science and technology studies, all of them working in or about brain research. This is an interesting contrast. One could argue that the more brain science is conducted in a technical milieu, as in neuro-technology, the more men/fewer women are involved. In this paper, I will not be addressing the perspective of women in neuroscientific research or neuro-technological development, but I was struck by the lack of females and Non-whites^5^ in the ARTE documentary. One might suspect that underlying gendered and racist concepts of the field invite or deter researchers in line with their suitability with regard to its objectives. Instead, I will focus on the neoliberal and colonial embeddedness of transhumanist visions, that, as Francesca Ferrando argues, is targeted at particular (upper) classes with economic power and consequently encodes racial and sexual politics (Ferrando, 2013, p. 27). Moreover, transhumanist visions and developments “realize the disembodied human self of the Enlightenment, purified and enhanced by science, medicine, and technology […] a super-human dream of perfection as an infinitude that harbors a disregard of vulnerability” (Åsberg and Nematidis, 2013). In the ARTE documentary, Miguel Nicolelis, a leading developer of neuro-technologies, described the field as a European, US, and Japanese endeavor, thus placing it within the framework of North-Western dominance. He neglected to mention any other part of the world.

The two aspects of the documentary, i.e., the “new” intelligible connection between neuroscience/neuro-technologies *and* transhumanist visions along with the intersected ascriptions within these fields of research, developments, and visions, are the topics of my paper. My research has already addressed the impacts of gendered concepts within neuroscience and neuro-technologies, their grounding in Western neoliberal socio-cultures and, vice versa, their contribution to the persistence of powerful gendered hierarchies and discriminations (Schmitz, 2012, 2016, 2017). I will augment my analyses in this paper by looking through an *intersectional lens at the gendered and racist notions which frame BCI research and development, culminating in visons of brain emulation, and at what outcomes are intended for whom*. In particular, I will examine the prominent focus on the enhancement of a masculinized, *white* rationality and efficiency, while ignoring a feminized, uncivilized emotionality, drawing on the long herstory of Feminist Science Studies and the latest concepts of Postcolonial Feminist Science Technology Studies (for in-depth analyses, see Pollock and Subramaniam, 2017; Subramaniam and Willey, 2017; Subramaniam et al., 2017). The last of these uncovers the sexist and racist concepts of rationality and civilization vs. emotionality and uncivilized *otherness* as a product of Enlightenment in coalition with colonial politics. Another revelation from the ARTE documentary was that I have “encountered” most of the neuro-protagonists during my research on BCI developments over the last 15 years. This clearly raises questions about the mutual roots of this (re-)union. My second focus of the paper, therefore, aims to *identify the roots within neuroscientific research and neuro-technological developments that may lead to legitimization of transhumanist visions*. However, in both analyses, I will also *search for the inconsistencies and ruptures* that contradict the straight lines of intersected ascriptions contributing to discriminatory outcomes. How does (feminized) affect as compared to (masculinized) rationality come into play in neuro-technologies and transhumanist visions? What kind of obstinacy characterizes the BCI and what “trickster” (Haraway, 1992) qualities thwart the *white* neoliberal story of ultra(trans)humanism? With this approach, my third aim is to challenge the intersectional taming of neuro-technological realizations and transhumanist visions to *formulate a concept of neuro-posthumanities* that could be realized *in other ways* than by targeting heteronormative and intersectional “-isms.”

Before starting this analysis, I will briefly describe my standpoint to the field of BCI-to-transhumanist visions. Fascination and apprehension accompany the development of brain technologies from Brain–Computer Interfaces (BCIs^6^, for the enabling of impaired patients) to Brain-to-Brain Interfaces (BTBIs, fostering direct communication between brains). On the one hand, neuro-technologies can and should help humans in the case of illness or disease: for facilitating communication with ALS patients (amyotrophic lateral sclerosis, a neuronal disease in which a patient progressively loses muscle control and thus the ability to communicate), for the rehabilitation of mobility after a stroke or provision of neuro-prostheses, or for regulating symptoms of Parkinson disease using deep brain implants. On the other hand, these developments also provoke fears, ranging from possible uncontrollable effects on body, mind, or surroundings, ethical aspects of ownership, and the risk of neuro-prostheses injuring self or others. Furthermore, these ambivalences increase when it comes to debates about the potential of superseding “human nature” with neuro-technologies in transhumanist visions (Schmitz, 2017). My own ambivalence concerning neuro-technological phenomena is *not* about their possible realizations going beyond human “nature.” If technologized brainbodies materialize through continuous intra-actions, and if they constitute and constantly re-constitute in science, technology, and society, these cyborgs, as Haraway (1985) has argued, may bear the potential to disrupt the modern dichotomy between feminized nature and masculinized culture (with technology seen as part of culture). As such, TechnoBrainBodies-in-Cultures (as I call them) could make reductionist biological determinisms obsolete, particularly those of rationalized masculinity vs. affective femineity that are used again and again to legitimize gendered and intersected lines of difference, social orders, and norms. However, the cyborg metaphor is ambiguous, oscillating between the potential of imploding binary orders on the one hand and the horror of control and exploitation on the other. Haraway, in most of her *Cyborg Manifesto*, has already emphasized the powerful practice of domination through informatics that legitimizes intersectional inclusions and exclusions from citizenship (Haraway, 1985).

My understanding of TechnoBrainBodies-in-Cultures is based on politically framed feminist materialisms as onto-epistemological phenomena, embedded in time–space undergoing a process of constant change. I employ Karen Barad's agential realism (Barad, 2003, 2007) as an analytical perspective to consider the agential forces of matter, technologies, and creation of meaning in BCI. I understand the notion of agency as an enactment that is not necessarily bound to consciousness or intentionality, attributes that are commonly aligned to human subjectivity. Agency in this sense

“*is the enactment of iterative changes to particular practices—iterative reconfigurings of topological manifolds of spacetimematter relations—through the dynamics of intra-activity. Agency is about changing possibilities of change entailed in reconfiguring material-discursive apparatuses of bodily production, including the boundary articulations and exclusions that are marked by those practices in the enactment of a causal structure” (Barad*, *2007**, p. 178, italics taken from the original)*.

Diana Coole's concept of politically framed materialisms complements my theoretical framework,

“in order to understand its materialization and, from a critical perspective, the way it is entangled with power relations, it must attend to the microscopic and macroscopic, the molecular and the molar. This means tracing politico-economic, geopolitical and biophysical circuits, conduits and networks through which matter passes as it is transformed, given surplus value, degraded, rerouted, hoarded and so on” (Coole, 2013, p. 464).

From the perspective of political feminist materialisms, the field of Brain(Human)–Computer (Techno)–Intra-actions touches on a range of questions concerning the agencies within these phenomena, the transgressions of subject–object and culture–nature boundaries through their realizations, their impact within bio-techno-socio-cultural entanglement, as well as their intersectional taming.

My following analysis will be undertaken in three steps. *First*, I will enroll the visions of transhumanism which “aim[s] to uphold the energy and political might of millions of transhumanist advocates out there who desire to use science and technology to significantly improve their lives”^7^. This agenda obviously raises the question of whose brains and lives should be improved and whose should be excluded from its visions (Hughes et al., 2016). Competition turns out to be defined as the normative capacity for the visions of uploading the mind to silicon: “[c]ompetition is an inescapable occurrence in the animate and even in the inanimate universe. To give our minds the flexibility to transfer and to operate in different substrates bestows upon our species the most important competitive advantage^8^.” I will take up some of the underlying concepts of transhumanist visions and work out the depth of their framing by intersectional hierarchized categorizations in terms of what should be technologically enhanced in what ways, by whom and for whom. I will not analyze the whole framework of transhumanist singularities (for details of the multiple fields, see Ferrando, 2019, p. 29–38; Gladden, 2018) but focus on those lines of arguments that intersect with recent brain science and neuro-technological developments, particularly when improvements shift “closer to transhumanist-impelled ideas in the field of neuroscientific brain research that focus rather on enhancement than on treatment” (Stollfuß, 2014, p. 92). According to my particular perspective, it would be important for these facets to be disclosed when it comes to the framing of TechnoBrainBodies-in-Cultures for a neoliberal, *white* masculinized effective North-Western society.

In a second step, I will question how the concepts of BCI developments frame the discourses on mind upload in transhumanist visions—or vice versa, how the latter frame the former. Over the past decade, there have been some analyses of transhumanist trajectories (e.g., Sharon, 2012; Ferrando, 2019), of how neuroscience and transhumanism interact (e.g., Stollfuß, 2014)^9^, and of intersected inscriptions in transhumanism (e.g., Åsberg and Braidotti, 2018). However, there is a dearth of analysis about how gendered and intersected inscriptions are embedded in neuro-theories and BCI developments and how they are taken to legitimize visions of enhanced artificial brains or ultra-humans. I will ask how neuro-technological developments strengthen or transgress gendered and racialized intersectional inscriptions, whereby masculinized thought and rationality is the to-be-enhanced vs. feminized emotions and unconsciousness is the to-be-avoided. Particularly, I aim to search for fractures that could lead to the inclusion of *otherness* and thus to alternative perceptions of TechnoBrainBodies-in-Cultures.

In a *third* step, I outline the current embedding of neuro-technological developments within a normative neuro-governmentality of enhancement. Based on my previous analyses, I will challenge the term “transhumanism” by contrasting it with the term “posthuman*ities*,” for which I am indebted to Cecilia Åsberg and the Posthumanities Hub; this is an approach that aims to question the more-than-human condition with the help of inventive feminist materialist philosophies. I aim to question how neuro-posthuman*ities* could be realized *in other ways* than by targeting heteronormative and intersectional “-isms” (as in transhumanism). Thus, I hope to develop strategies to integrate into cyborgian developments of TechnoBrainBodies-in-Cultures all the “disturbing intersectional stuff” of affect, sociality, aging and dis/abilities, uncontrollable bodily agencies, as well as obstinate agencies and contumacies. This will be not only an analysis but also a feminist intersectional future perspective.

The following analysis is not rectilinear. It will unfold in loops and sidetracks to uncover not only intersectional issues but also incongruities showing that these developments are not as easy and (pre)determined as expected, a journey through and with TechnoBrainBodies-in-Cultures that hopefully will open up other interpretations for posthuman*ities*.

## Transhumanist Visions: Brain Upload—Whose Brains, Which Capacities?

I start with some clarification of terms, particularly those of posthumanism and transhumanism and the relations between the both. The main objective of the posthumanist agenda is to decenter the notion of the human in worldly phenomena. The term posthumanism, rooted in postmodernism and having evolved out of a philosophical, cultural, and critical agenda (Ferrando, 2019, p. 1), became prominent with the turn of the millennium and covers a two-fold approach: to acknowledge that the human's “imbrication in technical, medical, informatic, and economic networks is increasingly impossible to ignore” (Wolfe, 2009, p. xvi) and, at the same time, to unfold the impacts and effects of this biotechnological and techno-cultural entanglement on human concepts and identities.

In contrast, transhumanism is characterized as targeting “the enhancement of ‘human nature' with the help of advanced technologies such as nanotechnology, biotechnology, robotics and information and communications technology” (Stollfuß, 2014, p. 83). As such, it can be separated from the critical stance of posthumanism in view of its techno-reductionism as “a hierarchical project, based on rational thought, driven toward progression” (Ferrando, 2013, p. 28). While undoubtedly, posthumanist scholars have distanced themselves from the transhumanist visions of downloading or uploading the human mind to artificial hardware, there is a danger in today's reception of both terms. Post- and transhumanism, respectively, become mixed up and either term may be used solely to describe the enhancement endeavor targeting the more-than-human entities, particularly within the “populist strand of posthumanism” (Ginn, 2017, p. 3). To face this problem, Åsberg (2013) proposed a change in the terminology to the notion of posthuman*ities*, aiming at taking up the decentering prospect of posthumanism while sharpening its separation from transhumanism.

However, to provide a short impression of the critical posthumanist-to-posthuman*ities* agenda, I will outline a short herstory by following the conceptualization of the posthumanist perspective with focus on its particular facets regarding my paper. I start with Katherine Hayles' seminal book *How We Became Posthuman (1999)* in that she claims that posthumanism

“embraces the possibilities of information technologies without being seduced by fantasies of unlimited power and disembodied immortality, that recognizes and celebrates finitude as a condition of human being, and that understands human life is embedded in a material world of great complexity, one on which we depend for our continued survival.” (Hayles, 1999, p. 5)

Hayles, as well as other authors, revises the critical posthumanist approach to Haraway's Cyborg vision (1985), with the cyborgian concept of bio-techno entanglements as fact and fiction. Cyborgian realizations are already part of our world and the cyborgian concept holds out a vision of transgressing gendered binaries of nature vs. culture. The latter could potentially prepare the ground for naturecultures (Haraway, 2003) that might dissolve powerful intersected categorizations and discriminations. Haraway's feminist posthumanist approach has been taken up within feminist materialisms as a prolongation of feminist poststructuralism (Butler, 1990), meanwhile acknowledging the material–discursive entanglements within the becomings (Haraway, 2008) of worldly phenomena. Onto-epistemological analyses of scientific knowledge production and of bio-technological developments—all embedded in, impacted by, and affecting socio-cultural power relations—could lead, according to Barad (2007), to posthumanist performativity. These approaches have drawn a clear connection between posthumanist critiques and degendering objectives, as implemented by Rosi Braidotty in *The Posthuman* with her call for a decline “of secular scientific rationality allegedly aimed at the perfectibility of ‘Man”' (Braidotty, 2013, p. 37). Of importance for the focus of my paper is the intersectional lens of posthumanism that Josef Barla perfectly elucidates in an abstract for a seminar:

“Contesting the very dichotomy of culture and nature, ‘we' and ‘them', humans and nonhumans, feminist and postcolonial scholars emphasized the existential need for decentering and deconstructing the anthropocentrism, essentialism, and universalism inherent to Enlightenment humanism. Shifting the focus to the marginalized and marked—that is, to ‘all constituted as others, whose task is to mirror the self', as Donna Haraway put it—posthumanist theories aim for novel critical figures and tropes in a world thoroughly transformed by technobiopower and the technosciences. At the same time, transhumanism gains a foothold as a kind of technophilic hyper-humanism that seeks to take control over human evolution itself through the means of existing and hypothetical future technologies such as cognition enhancing drugs, nanotechnologies, cryotechnologies, and whole brain emulation.” (Barla, 2019)

I agree with these trajectories. Responding to the aspiration “toward elaborating alternative ways of conceptualizing the human subject” (Braidotty, 2013, p. 37) and developing modes for “continued survival” (Hayles, 1999, p. 5) with cyborgian visions (Haraway, 1985), I appreciate the recent modification of the critical and intersectional term “posthumanism” to become “posthuman*ities*” (Åsberg and Braidotti, 2018; Braidotty, 2018). I will return to this perspective in the last step of my paper, but first I will focus on the relations between neuroscience, neuro-technologies, and the transhumanist discourse.

The second (EU-based) *Human Brain Project*, conceptualized 2013–2023^10^, with a funding of 406 million Euros already up to 2020 (HBP Project Grant Structure, Web^11^), targets in the first instance at the improvement in the information exchange and networking between neuroscientific research groups and at sharing brain data with the help of neuroinformatics. Brain Simulation (i.e., the replication of brain architecture and activity on super-computers) appears as the second step in the HBP (Overview, Web)^12^, introduced with the phrase: “Can you imagine a brain and its workings being replicated on a computer? That is what the Brain Simulation Platform (BSP) aims to do” (HBP Brain Simulation, Web^13^).

The first HBP^14^ was already based on the notion of the “cerebral subject” (Ortega and Vidal, 2007), the anthropological figure of the human, according to which all decisions and actions are explainable and predictable from the brain. The second HBP as well is presented as the endeavor to research and collect comprehensive knowledge of the brain to explain all thinking and behavior of the human subject. Sven Stollfuß showed how the new facets of the EU-HBP are related to information technologies to “define the computational principles of the functional and structural organization of the brain” (Stollfuß, 2014, p. 84) with the enhancement-based notion of an “ICT-accelerated “in silico cerebral subject”” (Stollfuß, 2014, p. 91, italics taken from the original. Moreover, the prospects of the HBP extend far beyond the cyborgian individual. Neuromorphic technologies are targeted to implement biological neural networks as analog or digital copies on electronic circuits as SpiNNaker and BrainScaleS architecture. These trajectories exhibit a double feature, combining visions of brain upload with the aim of improving computer technologies based on the model of the brain:

“In the medium term we may expect neuromorphic technologies to deliver a range of applications more efficiently than conventional computers, for example to deliver speech and image recognition capabilities in smart phones. […] In the long term there is the prospect of using neuromorphic technology to integrate energy-efficient intelligent cognitive functions into a wide range of consumer and business products, from driverless cars to domestic robots. […] The fact that major companies like IBM have defined cognitive computing as their main business for the future makes the development of neuromorphic hardware architectures especially interesting and economically attractive.” (HBP, Silicon Brains, Web^15^)

These trajectories demonstrate even more strongly: the embedding of the “*in silico* cerebral subject” within neoliberal governance of enhancement, as well as the formation and perpetuation of those social structures based on the paradigm of neoliberal economic growth in particular.

A very controversial discussion within the heart of the European neuroscientific community frames the scientific policies in handling this project^16^. There have been various critical analyses of the “new” neuroscience conceptualizations (e.g., Choudhury and Slaby, 2012) and of the relationships between neuroscience, neuro-technologies, and neuro-governmentality (Maasen and Sutter, 2007; Rose, 2012). However, here I will concentrate on the prospected line of creating the virtual brain, and even of perhaps making the individual brain immortal in digital worlds. Stollfuß (2014) has impressively investigated the amalgamating trajectories of the HBP with reference to the transhumanist prognosis. In a detailed analysis, he draws parallels between the HBP project lines for collecting all brain knowledge in multilevel brain models with the help of brain simulation and supercomputers along the developmental lines in transhumanist concepts: drawing on such brain databases, these lines target functional brain emulation to species generic brain emulation, i.e., the setup of generalized brain surrogates in silicon. Moreover, individual brain emulation comprises three possibilities of “Whole Brain Emulation”: social role-fit emulation, mind emulation, and personal identity emulation, thus not only figuring out technological “thought” upload as seemingly personal decision but also fixing normative social roles and requested identity formations. Stollfuß adopts these transhumanist lines from some of the main protagonists in the transhumanist field, namely, Sandberg and Bostrom (2008), Koene (2013), and More and Vita-More (2013):

“To push the vision further, in the ‘century of the brain' the ICT-accelerated ‘*in silico* cerebral subject' in computational neuroscience—and particularly in the ‘Human Brain Project'—can easily be synchronized with the requirements of its media technological environment. In this point of view, the ‘Human Brain Project' moves closer to transhumanist-impelled ideas in the field of neuroscientific brain research that focus rather on enhancement than on treatment.” (Stollfuß, 2014, p. 92/92)

As such, the transhumanist visions of a “radical transformation of the human condition by existing, emerging, and speculative technologies (as in the case of regenerative medicine, radical life extension, mind uploading, and cryonics)” (Ferrando, 2019, p. 3) are being debated. What is missing to date, however, is a profound analysis of the newest lines in BCI development concerning its intersectional inscriptions in relation to transhumanism. I have shown already the heteronormative framing of targets of rationality and consciousness that guide (self-)technologies of cyborgian brainbodies, for example, the permanency of masculinized rationality as the to-be-enhanced and feminized emotionality as being ignored (Schmitz, 2012, 2016). Can I find new challenges or also new possibilities when looking at the latest developments and discourse?

## Roots and Ruptures in Neuroscience and Neuro-Technologies

First, a deeper probe of the concepts underlying neuro-technological developments and transhumanist visions is necessary. During the last decade, the imaging of brain's connectome has become the leading target at the heart of the new Human Brain Project to research and extract brain-based explanations of most human behavior. The leading slogan “We are our brains,” explicated by Ortega and Vidal (2007) has turned into “We are our connectome,” as Nicolelis phrased in the ARTE documentary. Moreover, the connectome is taken as an epistemic object (Rheinberger, 1997) to predict a future in which neuroscientific research will even be able to measure “thoughts,” anticipating a future-oriented ability to capture thoughts through technology.

At the same time, the development of brain structure, physiological processes, and activation networks turns out to be embedded in a constructive process operating between nature and culture. In principle, the plasticity concept can help to explain inter-individual diversity as well as intra-individual variability. Based on the concept of entanglement (Rippon et al., 2014), plasticity deconstructs essentialist and binary ascriptions to a sexed brain. However, the idea of plasticity and modifiability of the brain can go hand-in-hand with the “modern neurobiological determinism” (Schmitz, 2012, p. 262). This notion is used to predict human thinking and action from brain data at the time of measuring, independently of the emergent bio-socio-cultural plasticity. In consequence, the brainbody is still framed as the essential entity, as the origin and cause of behavior, cognition, and decision-making. I termed this neurobiological determinism “modern” in the true sense of the enduring Cartesian dualism (of nature vs. culture) with all its associated sexisms, racisms, etc. I have also shown that modern neurobiological determinism does not contradict trajectories of modification of the underlying neuro-materiality, but is almost always conducted in a controllable manner. Moreover, narratives in a neo-capitalist society have a tendency to align the brain's plastic capacity to the corresponding ideal of an adaptive and flexible subject (Schmitz, 2012, p. 262).

Meanwhile, brain images, brain imaginaries, and the concept of brain plasticity form the core of developments in new lines of neuro-technologies. Furthermore, transhumanist imaginaries of mind upload are legitimated by and depend on this view of biomatter-based full coverage of thinking and acting. The narrative of the brain connectome is also the starting point of the ARTE documentary (Denjean, 2017) as the most promising resource for upcoming future technologies enabling brain upload. The aforementioned Ken Hayworth argues that the brain is a program covering our identity (even our soul as he terms it), with our experiences saved in the brain's connections. Note that this model comprises within its neuro-determinist concept the bio-social becoming of the brain.

However, the brain's complexity (particularly that of the human, but even that of animal brains) enables processes of cognitive abstraction (“thoughts”) beyond neuro-materialism. Abstraction has neuronal correlates, but abstraction cannot be traced back to its origins in neuronal or connectome materiality, and respective neuronal activity alone. The brain's capacity derives from passing a threshold of complexity to achieve a more-than-material emergence. Emergence, as I learned back in the 1980s from my neurobiological mentor, does not mean something mystical. Emergence is a qualitative outcome of high complexity *per se*. Elisabeth Wilson in 1998 already referred to this aspect in her book *Neural Geographies*, where she elucidated the concepts of neuro-constructivism, stating that “figure cognitive processing as the spread of activation across a network of interconnected, neuron-like units” and “individual units have no representational status as such, it is the overall pattern of activity across the network in total [that counts]” (Wilson, 1998, p. 156). If, in principle, one is dubious about the possibility of a complete mind upload to silicon in view of its inherent more-than-material quality, this points to uncertainties about the explanatory value of neuroscience and poses questions about uncontrollable obstinacies within neuro-technological developments. As Alaimo (2014) states, these uncertainties and obstinacies challenge notions about the meaning of human subjectivity within transhumanist visions of mind upload, to say the least. This also calls for a speculative turn within the debates about feminist materialist performativity (Åsberg et al., 2015) and, in my view, calls for the opening up of the feminist materialist debate concerning the neurosciences and transhumanism to an integration of moments of fluidity, intersectional facets, and postcolonial movements.

I have analyzed BCI developments from around the turn of the millennium and in the first decade of the 20th century (Schmitz, 2012, 2016). In search of the foundations that lead to transhumanism, I will refer to these findings and proceed to focus on the relevant aspects of recent BCI research and development from the second half of the 2010s in order to search for intersectional issues that link various “-isms” such as rationalism, sexisms, racisms, emotionalism, controlism, and agentialism, with a particular focus on unforeseeable developments. In particular, this means tackling the frictions between rationality and affect (or better to say between thoughts and emotions), between controllability and obstinate BCI agencies, and to follow the material traces, actor's status, agency, and subject–object relations in closed and open neuro-technologies. All of these facets and their conceptions in BCI research and development are deeply intertwined. I am seeking to understand how they prepare the ground for the legitimation of intelligible targets in transhumanist visions, as well as how they are impacted by these visions. I search for their grounding in modern neurobiological determinism and for intersected norms and values that are inscribed therein, as well as for the fractures and discrepancies that may open up alternative views of a posthuman*ities* future. The following main protagonists all appear in the ARTE documentary (Denjean, 2017) connecting transhumanism with the latest neuroscience research and neuro-technological developments.

### Unconsciousness and Affect in BCI

A short review of the herstory of BCIs. In 1999, Nils Birbaumer (first big name in the play) and his research group presented an ALS patient who learned to change his EEG waves to move a cursor up and down on a computer screen in order to select letters. The researchers called this BCI initially the *Thought-Translation Device*, and under this name, it was widely disseminated and referenced. It turned out, however, that the successful realization of this BCI communication was not simply based on conscious decisions made by the patient but required processes of operant conditioning^17^. With this, unconscious facets also come to the fore and this unconsciousness is related to affective status. This most interesting aspect arises from the patient's “descriptions” with the help of the BCI system about his long-lasting efforts to produce a feeling of a “pressure in the brain” in order to select a letter or, alternatively, to “empty his thoughts” to achieve a letter rejection. This is how the PhD student, Nicola Neumann, who conducted the study, interpreted this practice:

“… it was not the controllable production of his ‘thoughts' (as a metaphor of the rational mind) but the inseparable entanglement with brain activities and even with sudden emotions that guided the communication process” (Neumann, 2001, p. 62).

However, this interpretation was at first only mentioned by psychology student Nicola Neumann in her thesis; in the subsequent publication by the whole Birbaumer group (Kübler et al., 2001), this focus on unconscious emotions instead of thought translation vanished when it came to presenting the findings from more patients.

Follow-up BCI developments addressed processes of conditioning to improve the communication between a brainbody and a computer, for example, to repair or replace damaged motoric brain areas in stroke patients. For my focus here, the newer developments that put affective stimulation into practice to develop even more effective BCI are worthy of note. Exhibiting a so-called *neuro-force feedback*, Silvoni et al. (2011) stimulated muscles of a paralyzed finger (unconditioned stimulus) and brain areas neighboring the stroke area (conditioned stimulus) to induce a plastic adoption of the finger's movement regulation by “new” brain areas. Castermans et al. (2014) operated a classical conditioning and combined muscle stimulation of a paralyzed limb with an activation of the motor cortex by a neuro-prothesis instead of conscious “thought” regulation. The transfer of the conditioning process into the BCI should “promote neuroplasticity in combination of traditional physiotherapy [bottom up] and robot-aided therapy [top down]” (Castermans et al., 2014, p. 34).

For completely locked-in patients, it is not clear whether they can understand a question if they cannot give an answer. De Massari et al. (2013) used classic conditioning to stimulate sensoric brain areas (unconditioned stimulus) simultaneously with questions and Yes/No answers about the name or mood (conditioned stimuli) of such patients. After 3 weeks of training, one patient showed some brain activation in response to the conditioned stimulus only. One might regard this as being only a marginal aspect of BCI development. Yet, the affective unconscious learning approaches were celebrated as a milestone in the further development of BCI to facilitate communication with completely locked-in patients (Chaudhadry et al., 2017). The Birbaumer group, in 2019, claimed that their studies would enable the examination of severely disabled patients in their domestic environment. These studies add value to mood-driven “communication,” at least for health issues.

Returning to the question of thought translation as a committed prerequisite for brain upload: it is interesting that the popular media prioritized another aspect: “Could Birbaumer read thoughts?” was the question posed by the German newspaper *Süddeutsche Zeitung* (Bauer et al., 2019). Although the Birbaumer group denied that they had been able to read thoughts with their studies, using brain signals alone to move a cursor or recording brain reactions to Yes/No answers, it is interesting how connections between measurement and thought translation were immediately drawn^18^.

In the past 2 years, however, emotion and mood-related recognition of communicative signals in the brain have gained more and more prominence in BCI research and development. The debate about the possibility of detecting “consciousness” in locked-in ALS patients or patients in vegetative state (VS) or minimal consciousness state (MCS) is gathering pace. Successful communication with three of eight patients in MCS has been reported with the help of EEG-based BCIs and by using movie clips of crying or laughing (Pan et al., 2018). The group released another publication in 2020, in which they reported an improvement of behavioral answers using an EEG-based BCI in 15 of 18 patients with cognitive motor dissociation (83.33%), whereas only 5 of 27 unresponsive wakefulness syndrome patients (18.52%) regained consciousness (Pan et al., 2020). Some BCI developers target EEG-based BCI as the technology of the future (Guger et al., 2017), while others are moving onto new technologies, e.g., time-resolved functional near-infrared spectroscopy (TR-fNIRS) based BCIs that detect the mean time-of-flight of photons to calculate the increase in blood oxygen levels in activated parts of the brain over time (Owen et al., 2006). The group of Adrian Owen was celebrated in 2020 for having extracted features of activity in the brain in 21 healthy subjects who “answered” a series of questions by imagining playing tennis for “yes” and staying relaxed for “no” (Abdalmalak et al., 2020). In this context, the efforts of the Owens group are interesting: to link non-purposeful imaginings (playing tennis) or mood reactions (staying relaxed) to consciousness is a similar project to the Birbaumer's group's first Thought Translation Device from 1999. Although this BCI has only been tested with healthy subjects up to now, it is prognosticated as an upcoming device for unconscious patients (Owen, 2020).

From an intersectional perspective, the acknowledgment of mood and affect as being important communicative facets sounds promising, alongside a vision of up-valuing these qualities of a more than solely rational mind. I argue that a focus on unconscious facets, mood, and affect could form the ground for a speculative turn on what could also be at stake in the development of neuro-technologies, besides the goal of thought translation and communicative enhancement. My approach draws a connection between decolonial and posthumanist agendas with respect to language, following Sousa and Pessoa (2019). This is a necessary backdrop for an informed discussion of the potential of up-valuing emotion within BCI development. In accordance with Mignolo (2018), Sousa and Pessoa criticize the dominance of seemingly unique principles of knowledge, challenging the concept of the operation of linguistic rules for maintaining homogeneity, normativity, and control in a Western agenda. Instead, they argue for recognizing decolonial concepts of language. This involves taking into account indigenous notions, e.g., of language connected to land, and elevating these to the same explanatory level as applied to knowledge. This decentralization would undermine the Western notion of lingual superiority. Sousa and Pessoa also point out that “[t]his colonial and humanist project focused on the idea of language taking place exclusively between human heads, and that entailed the disregard of people's bodies and senses” (Sousa and Pessoa, 2019, p. 531). Applying this approach to BCI communication, the valuing of emotions could decentralize the notion of (only) thoughts as the respected quality therein.

However, what becomes evident is a twist in the media narratives that turns mood-associated BCIs into a means of targeting the speaking capacities of the related (imagined) patients. I would argue that mood and affect, in these narratives, are not valued as qualities per se, but are only factors on the way to thought detection and rational conversation. The Owen group argues: “Basically, a brain-computer interface can read brain activity and find patterns for different words. In a way, the computer can speak for the person, only by connecting to the brain^19^,” or “BCIs are devices that allow the brain to communicate with an external device that ‘speaks' for them^20^.” Furthermore, in the scientific sphere, these developments refer back to a long-lasting debate about identifying the neural correlates that would be minimally sufficient for consciousness (Owen and Guta, 2019). Additionally, several lines emerge from this research that lead to transhuman features and neoliberal governance of emotion: “emotions research has a wide range of benefits from improving learning outcomes and experience in Intelligent Tutoring System (ITS), as well as increasing operation and work productivity” write Xu et al. (2018). There is an increasing market for BCI emotion recognition systems^21^ outside the health sector, most claiming to improve the individual management of work performance. Steffen Steinert and Orsolya Friedrich, in their paper on ethical issues of upcoming affective BCI, give an overview of devices that are able not only to detect but also to influence and stimulate affective states. “For example, emotional profile building could help to subtly emotionally influence people for economic or political gain. Due to the sensitive nature of data about mental states, issues of mental privacy, cognitive liberty and mental integrity have to be raised with stronger emphasis” (Steinert and Friedrich, 2020, p. 363).

### Obstinacy in the Closed Loop

Miguel Nicolelis and Michael Lebedev (the second group of big names) have staked out the ground and claimed the territory in neuro-prosthesis development with their Macaques study. Again, a little herstory: Aurora (sic!), a young female macaque, learned to move a ball on a computer screen with a joystick into a cube. Along the way, a parallel control of a robotic arm was conducted with the same action. When the researchers removed the joystick, the ape decreased her arm and hand movements. However, the movement of the robotic arm continued. The authors concluded that the ape learned to operate the robotic arm solely by neuronal activity; by her thoughts, as they first framed it (Nicolelis, 2003). However, very soon, Lebedev and Nicolelis (2006) developed the term “closed loop.” The ape needed multiple feedbacks during training, i.e., food reward and visual and sensory feedback, to learn a successful regulation of the prosthetic arm. As in the case of the ALS patient in the first Birbaumer study (Birbaumer et al., 1999), a visual feedback from the moving cursor on the computer screen was essential as a positive reinforcement to acquire the skill to lower or raise his cortical potentials. Thus, an effective development of BCI and neuro-prostheses depends on the learning plastic brain and learnable algorithms, which mutually frame each other *inside* the bio-techno materiality. The term closed loop has entered the field of neuro-technological developments and is used widely, but it is by no means banal.

I have shown (Schmitz, 2016) how, in the following years, the responsibility for the learning process and plastic reorganization within the brain was successively assigned to the technology. The legitimation for this handing over the signal responsibility to the technological agency was its higher efficiency in rehabilitation. Cunningham et al. (2011) did not themselves program the algorithms for neuro-prostheses but let them calibrate “*online*” with the brain to improve the algorithms for neuro-prosthetic control. Such an *Online Prosthesis Simulator* (OPS) proved to be more accurate without the intervention of the developers (for details, see Schmitz, 2017). Similarly, in a *functional electrical stimulation* (FES)-BCI (Soekadar et al., 2015), the conditioned stimulus that regulated a movement of a robotic finger was generated from the algorithms and not by the developers. Not only should the brain learn due to its plasticity, the software algorithms of the neuro-prosthesis too should adapt gradually to the brainbodies' rehabilitation process.

Nevertheless, with the closed loop, neuro-prosthetic development has been assigned a unique ontological status. The concept acknowledges a kind of obstinate agency within the BCI, in the sense of mutual learning and formative processes between the brain and the technology. I would speak of an *obstinate agency* within the closed loop that could offer unforeseeable phenomenal becomings. What could it mean when arm amputees learn impossible arm movements while observing an artificial arm and imagining “impossible” movements of the phantom arm (e.g., bending the forearm against the elbow), perhaps even developing a self-schema and a feeling of ownership of this impossible arm (Moseley and Brugger, 2009)? The artist Stellarc played with such (im)possible cyborgian developments, by connecting a third arm to his body^22^, for example, which also could be regulated from someone somewhere else via an internet connection. Stellarc related his performances explicitly to Haraway's cyborg vision of transgressing nature-techno boundaries alongside gender binaries (Hunt, 2015).

However, BCI researchers and developers do not name this obstinate material agency explicitly. This is my terminology only, drawing on the feminist materialist framework. For the developers, a certain disposal of control in online programming within the neuro-prosthesis seems to be indispensable. In BCI development, a freedom of learning should only be allowed if it is “effective but non-ambiguous” (Castermans et al., 2014, p. 35); it should improve the effectiveness of internal bio-technological interrelations, but maintain controllability. However, this raises the question of how much degree of freedom should be accorded to the BCI by this transfer. In the case of BCI development for rehabilitation of disabled functions due to age, disease, injury, or accidents, effectiveness *and* controllability of a BCI could be seen as legitimate and even essential. Unexpected bio-technological intra-actions in the closed loop could undermine rehabilitation and are to be avoided. Moreover, from a juristic point of view, the question of who is accountable is still not regulated: if, for example, a neuro-prothesis suddenly hits other people, is it the fault of the patient, the developer, the researcher or even the BCI itself, as (Clausen, 2006) asks?

For neuro-prosthetic developments, it is worth taking a look back at how it all started. The DARPA (the US Defense Advanced Research Projects Agency) financed most of the original research on the neuro-prosthetic development by Nikolelis and Lebedev. The Rehabilitation Institute of Chicago provided Jesse Sullivan, a double amputee from Tennessee, with two neuro-prostheses, regulated with nerve-muscle graft (Craelius, 2002). In 2005, he was presented as the “the World's First ‘Bionic Man”' (referring to a 1970s TV series *The Six Million Dollar Man*) and as the first non-fictional cyborg (RIC, 2005). Jesse Sullivan was followed by Claudia Mitchel, a female and Black former U.S. Marine Corps officer—the bionic woman—who could regulate her neuro-controlled prosthesis (bionic arm) “simply by thinking” (RIC, 2006). RIC reported on the case of Jesse Sullivan in BBC News: “In fact, we are actively engaged in a proposal process to revolutionize prosthetics with the Defense Advanced Research Projects Agency of the US Department of Defense^23^.” For example, the “Revolutionizing Prosthetics Program,” launched in 2007 and extended in 2009, aimed at enabling injured soldiers to control an artificial arm via neuronal interfaces. Rehabilitation and operation readiness are not clearly separable. In this domain, medical applications and non-medical techniques of optimization in individual and weapon development cannot be distinguished sharply (cf. Schmitz, 2012). Hoag (2003) characterized the military sector as taking a predominant role in financing the development of BCI, neuro-prosthesis, and further neuro-technologies (Gibbs, 2008) for the faster, harder, fit-for-action, always ready-for-operation soldier. In the ARTE documentary Denjean (2017) the development of exo-skeletons and exo-prostheses is also mentioned as the latest innovations by the Nicolelis group.

In conclusion, despite their prima facie application scenario for the treatment of dis/abilities, neuro-prosthetic BCI also serves effectiveness in an analogous manner to the economic evaluation of affective BCI. There are other scenarios that call for an intersectional discourse around neuro-prostheses. For example, elite sports exhibit a severe binary division, mostly dominated by notions of masculinized bodies as muscular, powerful, and competitive; female bodies have to adapt to these signs of masculinity (Harasser, 2013), and this is only permitted up to a particular threshold. If a body is found to have exceeded the accepted threshold, as in the case of Caster Semenya's body, it will be excluded. Indeed, media representations underline the exclusion from their imaging choreographies of Non-white bodies considered to have an unfair advantage (Kleindienst-Cachay and Heckemeyer, 2008). Furthermore, a debate about fairness arises when, for example, runners like Oscar Pistorius with double leg-prostheses call for permission to join the competition between healthy athletes. The debate advanced arguments to the effect that athletes benefitting from such technologically enhancement would have an unfair advantage in the competition. However, other notions in this debate highlight elite sports as a “critical transformative room” (Crutzen, 2016), as when pharmacologically or technologically enhanced athletes are perceived as being superhuman cyborgs. Not only can these subjects no longer serve as displaying signs of dis/ability, but they are even celebrated as “ambassadors of transhumanism, placed at the cutting edge of human boundaries of capability” (Miah, 2003).

On the other hand, the use of prostheses has also erased a further evaluation of dis/ability—albeit with ambivalent meaning-makings. Victoria Modesta, calling herself a “bionic pop artist,” deconstructs the notion of a leg prosthesis as a mark of dis/ability in her performance in “Prototype^24^” by acting with the prosthesis in various forms in a powerful and political scenario. Double leg amputee and top model Amie Mullins plays with up to 12 different pairs of prostheses on the cat walk as well as in artist performances; according to Garland-Thomson (2002), she creates an image of miraculous, sentimental, exotic, and realistic stereotypes in the popular media. A Syrian refugee, Ashraf Albesh, who was fitted with a prosthetic leg similar to those worn by Pistorius, “discovered his entanglement with the artificial leg as a means for dancing,” amounting to the “diffractive transformation […] into disturbance giving open space for new possibilities” (Schinzel, 2021). Knöppchen (2018) analyzed the campaign film *Die neue Nähe* [The New Proximity] made by the German dis/ability funding organization “Aktion Mensch.” Focusing on the bodies, interactions, and communication in these film sequences, she investigated the extent to which the revision of notions of the *otherness* of people is even possible—and found some playful “encounters” between the people with disabilities and children in the film shots. However, she also found that the boundaries and normative conformity of the interacting “abled” and “dis/abled” partners had been reestablished.

For me, the question remains open as to whether neuro-prosthetic development may offer the possibility of changing the evaluation of dis/ability and uncovering obstinacies within prosthetic cyborgs over and beyond the astonishment elicited by exotic examples, or whether its embedding in the effectivity discourse will prevail.

### Subject–Object Relations: The Machine Model

Remember, in the first BCI discourse, it was the human subject that was supposed to regulate the device with “thoughts.” However, if developers progressively target unconscious or affective stimulation and mutual learning through brain plasticity and learnable algorithms or predict BCI calibration more accurately and effectively without involvement of a programmer within the closed loop, one also could argue that the BCI itself achieves a type of actor status through its obstinate agency. If the BCI acts, and if bio-technological intra-actions change directions, agents, and recipients, does the bio-technological agency then challenge notions of the subject–object relationship? Referring to the central paradigm of critical posthumanism is the notion of the intentional acting human subject decentered within the mutual conditioning between brain, body, computer, and neuro-prosthesis.

My analyses of recent publications in the field show that instead of a concept of obstinate agency or even subjectivity, BCI are reformulated as a comprehensive machine model. Moreover, the machine model defines the ground for targeting material traces anywhere in the bios, the techno or the silicon. Patients' “decisions” are termed “plastic conditioned pattern of brain activity” as, for example, when Castermans et al. (2014) argue for rehabilitation with a *robot-aided* BCI based on a concept of the “human locomotion machinery” or when a limb representation is termed a “completely novel body image … constructed solely by internally generated mechanisms” by Moseley and Brugger (2009, p. 18798). Miguel Nikolelis uses the machine metaphor to defend BTBI developments as follows: “Basically, we are creating what I call an organic computer^25^.” Last but not least, in the ARTE documentary, Ken Hayworth, in two lengthy scenes at the beginning and the end of the video, claims that not only the brain, but also the “humans are programs.” My hypothesis is that, once again, the machine model is being promoted to maintain control. Thoughts, decisions, experience, and subjectivity are bio-materially coded in a machine model of the whole BCI, including the human and the technology. Based on this notion only, and taking up the analysis of Stollfuß (2014), the vision of “Whole Brain Emulation” can comprise social role-fit emulation, mind emulation, and personal identity emulation. The main purpose is to trace, but what exactly will be traced in what direction?

### Opening the Closed Loop

The primarily conceptualized *closed loop* between the brain and the algorithms opens up toward input from outside in so-called *Brain-to-Brain Interfaces* (BTBI). In 2013, for the first time, the Lebedev/Nicolelis group reported a transfer of sensoric information from a so-called “*encoder*” rat to a “*decoder*” rat via a BCI in order to let the latter select a stimulus (Pais-Vieira et al., 2013). One year later, the development of BTBI between humans was already envisioned along with virtual information transfer between human brains over long distances via the internet (Grau et al., 2014; Rajesh et al., 2014). Besides public celebration of this “feasibility of a biological computer consisting of a network of animal, or human brains” (Gorman, 2013), there was also doubt about the validity of the data on direct brain transfer (Cossins, 2013). Interestingly, I could not find newer publications on concrete developments of BTBI in humans.

Trimper et al. (2014) name ethically problematic aspects of possible BTBI, e.g., neural privacy or informed consent, ownership of one's own thoughts (do they belong to the transmitting brain or to the receiving brain?), and also data security in information transmission via internet and the protection against hacking, in military applications, for example, or impacting the receiving brain with traumatic memories.

Interestingly, the first BTBI publications have also been referenced in economic affairs newspapers^26^, hinting at the possible major targets of pathways within these developments. However, the visions of opening up the closed loop become evident in one of the most recent publications on *Human Brain/Cloud Interface* in *Frontiers in Neuroscience* (Martins et al., 2019). Here, the enhancement of human cognitive enhancement (referring to Kurzweil) as a prolongation of BCI and BTBI is anticipated. Based on the prospective application of nanorobots in the human brain, coined neuralnanorobotics, Martins et al. envision the development of a real-time interface between human brains and the internet via supercomputers and artificial intelligence algorithms, the B/CI, within the next 20–30 years. The concept is based on drawing a parallel between the “quantitative human brain,” imagined as a huge depot for information storage, and the “cloud,” i.e., the infinitive knowledge center in the internet. This project again builds on the neurobiologically determined neuroscientific model of the brain as connectome and an expected “non-destructive, real-time, secure, long-term, and virtually autonomous *in vivo* system” (Martins et al., 2019, p. 9, italics taken from the original): different types of neuralnanorobots implemented in the brain should enable it to be connected to the internet.

This amounts to the transformation of the vision of the “*in-silico* cerebral subject” proposed by Stollfuß (2014) into reality. The aligned prospects again reflect the colonial *white* Western masculinized notion of the to-be-enhanced. The targets of the B/CI are outlined explicitly, prompting several critical questions: “significant improvement in education” challenges what should be learned in future; “enhancement of human intelligence” puts into question the form of intelligence envisaged; “artificial intelligence and existential risk prevention” refers to the advantage of AI over human intelligence by putting language capacities (sic!) at the center; and, finally, “transparent shadowing” reaches out to the transhumanist vision of virtual twins mirroring the host's life experiences and acting as attendees in various settings. What the authors really mean by highlighting the virtual autonomy of their cyborgian developing in line with the promise of control (security) remains an open question. Could that allow the appropriation and application of unforeseeable knowledge and actions?

## Visions of Posthuman*ities*: Taking Up the Intersectional Stuff

I have found some roots in BCI/BTBI developments that show pathways to serve transhumanist visions of technological enhancement leading to future upload of the mind to silicon. I have also discussed some possible unforeseen trajectories that could transgress intersectional categorization of a hierarchized masculinized, *white* on-the-top rationality over a feminized, underdeveloped unconscious affect. The shift to measurement of unconsciousness could open up cyborgian development to an obstinate bio-technological agency with diverse degrees of freedom; emotion-targeted BCI could up-value affect as a significant aspect of communication. All these are practices of un-taming the intersectional inscriptions within BCI. However, in upcoming practice, BCI/BTBI are mostly designed for enhancing effectiveness and competition of the individual in society. Effectiveness within the closed loop is achieved through control, while the machine model serves the idea that the matter of tracing thoughts could be equally bio or techno or biotechno.

The transhumanist agenda is strongly associated with “-isms” that always encompass political aims and norms. I have tried to gain political momentum with feminist STS and feminist–materialist approaches with regard to BCI phenomena that are addressed in transhumanist discourses and applying concepts of neuro-governmentality. Referring back to my setup of the tension between a cyborgian potential to transgress gendered and intersected binaries, respectively, to deconstruct the discriminatory assignments thereof, and the danger of powerful practice of domination through informatics, I revise the development of the “*in-silico* cerebral subject” (Stollfuß, 2014) within socio-cultural power relations. In order to gain and fulfill biological citizenship (Rose and Novas, 2005), the Western dispositives of personalization, self-responsibility, and particular enhancement goals still legitimize social positioning and societal success (Pitts-Taylor, 2010; Schmitz, 2012). The combination of invocations demanding a self-responsible application of neuro-technological enhancement underpinned by the gendered and racialized ideologies and normative demands of current neuro-governmentality (Maasen and Sutter, 2007) point to the particular adoption of masculinized *white* norms and values in these developments.

What is happening today outside the health sector is the development of techno-human enhancements to achieve a spectrum of particular objectives, with controlling bodies and their capabilities within the work environment (Farah et al., 2004; Schmitz, 2012). Capitalist-compatible techno-enhanced physicality turns out more and more to be a critical success factor in the construction of identities and the profit-oriented marketing of one's own labor with flexibility, competitiveness, rational productivity, concentration, effectiveness, multitasking, and efficiency available on demand. Surgical techniques, Ritalin or Prozac intake, Brain Caps, or internet connections all increasingly intervene in the body, so that the postmodern *white* Western subject becomes caught in the wheels of improvement, expansion, and optimization under the slogan of “feasibility rather than fate.” Enhancement techniques for any kind of skills and moods based on notions of brain plasticity are promised for everyone (see, Greely et al., 2008 in *Nature*), seemingly regardless of gender. However, analyses from a feminist perspective have shown that gendered attributions are (again) produced in scientific and popular discourses concerning the applications and practices of neuro-enhancement (e.g., Blum and Stracuzzi, 2004; Höppner and Schmitz, 2014).

In addition, the repeated references to thoughts and minds as the driving forces of neuro-enhancement produce an image of the conscious and self-confident subject that uses the technologies for her/his own aims and needs. The apparently autonomous setting of human decision-making and controlled communication masks the embedding of these self-technologies in current neuro-governmental bio-politics and the interplay of research policies, markets, and state politics (Pickersgill, 2013). The other side of the coin is control: technologies for face recognition, border control against *others*, namely, Non-white immigrants that are not supposed to enter the North-Western sphere. Learnable algorithms that surf the internet promote prejudice Black people and women simply by taking up precisely what humans have posted (Noble, 2018). Racial profiling and predictive policing tools that link “places, events, and historical crime rates to predict where and when crimes are more likely to happen” generate sexist, racist, and classist discrimination (Heaven, 2020) and so forth. A lengthening of this list would go far beyond the scope of this paper. I have considered the military sector in relation to the aspect of control, because it plays a predominant role in financing the development of BCI, neuro-prosthesis, and further neuro-technologies (Hoag, 2003).

Despite this seemingly overwhelming continued predominance of colonial masculine power, it is important to formulate strategies for responsible and accountable research, development, dialogue, and discourse for the realization of TechnoBrainBodies-in-Cultures *in other ways*. Cecilia Åsberg's perspective of posthuman*ities, a* “philosophy and sciences informed by advanced cultural critique and some seriously humorous feminist creativity […] and inventive feminist materialist philosophies” aims at joining “postdisciplinary arts and sciences informed by cultural critique and feminist creativity” to research and at discussing “the more-than-human condition^27^.” With this perspective of posthuman*ities*, and trying to imagine speculative turns within the developments of BCI to mind upload, I have tried to challenge the term transhumanism and assess the impact of heteronormative and intersectional inscriptions in TechnoBrainBodies-in-Cultures, while at the same time trying to find even small windows of obstinacy, unforeseeable possibilities in development and reinterpretations of the meaning of what should be enhanced.

In my view, potential idiosyncratic practices within TechnoBrainBodies-in-Cultures, unforeseeable agencies, and an openness for the diversity of their realizations are more to be welcomed than feared. The point is that cyborgian developments, so far, have not realized uncontrollable autonomous agencies that could control or dominate humans or human societies. These are only fictional horror scenarios. Instead, it is the sphere of human developers and practices with their targets, concepts, and realizations in creating TechnoBrainBodies-in-Cultures that needs to be subjected to critical discourse in order to further an open and non-discriminatory posthuman*ities* debate. Following this approach obstinacies could also be envisioned for obstinacies could also be envisioned for cyborg futures, embracing components such as affect, sociality, contumacies, uncontrollable bodily agencies, as well as idiosyncratic agencies and contumacies. The term posthumani*ties* thus becomes clearly distinguished from transhumanism. One way would be to conduct an in-depth search for further examples of TechnoBrainBodies-in-Cultures realizations and trajectories that exhibit cultural and ethnic diversity, age, illness or dis/abilities, and multiple sexes/sexualities.

Another could be to encourage critical reflection of the field by the public and the academic world, namely, by developing neuro-literacy formats. Ashley Baccus-Clark, a molecular and cellular biologist, multidisciplinary artist, performer, writer, and “brand strategist,” works collectively in Hyphen-Labs with other women of color, “at the intersection of technology, art, science, and the future” (Hyphen-Labs, 2020), and develops academic-arts performances, e.g., NeuroSpeculative AfroFeminism, a virtual reality project that unfolds visions of neuroscience and neuro-technology in other ways (Baccus-Clark, 2020). As a result of the most recent conference of the NeuroGenderings network in Leiden 2020, a group of neurofeminist-arts scholars and myself have now initiated a working group for developing “neuro-literacy” at the intersection of STS and arts/performances.

This paper is a start and a call for NeuroGenderings scholars to join in a transdisciplinary working group for developing further analyses within a posthuman*ities* perspective. My vision is to regard TechnoBrainBodies-in-Cultures as cyborgian companion species instead of enhanced competitors (Haraway, 2003), with all their inexplicabilities, unpredictabilities and idiosyncrasies, vulnerabilities, and incompleteness, that could support us in challenging neurobiological determinism and anthropocentrism.

## Data Availability Statement

The original contributions presented in the study are included in the article, further inquiries can be directed to the corresponding author.

## Author Contributions

The author confirms being the sole contributor of this work and has approved it for publication.

## Conflict of Interest

The author declares that the research was conducted in the absence of any commercial or financial relationships that could be construed as a potential conflict of interest.

## Footnotes

^1^I have taken the ARTE documentary as a starting point and as a counterfoil throughout this paper, because it is one of the most recent documentaries in this sphere, along with others, and even more because ARTE is a TV station that is known for its critical features on science–societal relations and impacts.

^2^This field in brain science researches the overall network of fibers and connections within the brain: the connectome.

^3^https://www.brainpreservation.org/ (accessed January 6, 2021); the website also calls for donations, and in the ARTE documentary, it is told that Hayworth himself has already invested more than 1 million dollars in cryopreservation techniques and in the development of supercomputers to save (his) brain data.

^4^https://neurogenderings.wordpress.com/ (accessed January 6, 2021).

^5^This does not hold for the transhumanist movements whose internet presentations are more diverse with respect to gender and ethnicity of its members.

^6^In the following, I subsume Brain-Computer Interfaces and neuro-prostheses, the latter often termed as Brain–Machine Interfaces (BMI) under the one term of BCI.

^7^http://www.zoltanistvan.com/TranshumanistParty.html (accessed January 3, 2021); Zoltan Istevan in one of the founders of the Transhumanist party, and its today's political and Media Advisor, see http://transhumanist-party.org/advisors/.

^8^http://www.scifuture.org/extending-life-is-not-enough/ (accessed December 27, 2020).

^9^Sven Stollfuß analyses the EU Human Brain Project “in order to emphasize the rise of the posthuman brain on the backwash of visions between treatment and enhancement” (Stollfuß, 2014, p. 82).

^10^https://www.humanbrainproject.eu/ (accessed December 26, 2020); the HBP connects more than 110 European and international research institutes and companies, e.g., CoreTec with Pascal Fries as member of its advising board, mentioned here because Fries is also chair of the Ernst Strüngmann Instituts (ESI) for Neuroscience in cooperation with Max Planck Society, Frankfurt, and he was a protagonist in the ARTE documentary (Denjean, 2017).

^11^Human Brain Project Grant Structure, https://www.humanbrainproject.eu/en/about/human-brain-project-ec-grants/ (accessed December 26, 2020).

^12^https://www.humanbrainproject.eu/en/about/overview/ (accessed December 26, 2020).

^13^https://www.humanbrainproject.eu/en/brain-simulation/ (accessed December 26, 2020).

^14^The first HBP was funded by the US National Institutes of Health under the realm of the “Decade of the Brain” (1990–1999), proclaimed by George Bush. Its main objective was to sample brain data from the genetic up to the functional level of the brain, to develop tools for presenting brain data and for matching data between research groups (Koslow, 2000).

^15^https://www.humanbrainproject.eu/en/silicon-brains/ (accessed December 26, 2020).

^16^An Open Letter, signed by 156 “Principal Investigators/Directors, eligible for HBP funding” and addressing the European Commission was launched on July 7, 2014, on http://www.neurofuture.eu/ (a page that is not available anymore). Mostly scholars from cognitive neuroscience complained not only about the exclusive funding politics of the HPB but also about its plan to map the entire human brain in computer models (Editorial, 2014).

^17^Conditioning is a learning procedure, the term derived from animal behavioral studies, that is based on the materialization of timely associated inputs into the brain. Classical conditioning sets an unconditioned stimulus (i.e., food) simultaneously with a conditioned stimulus (i.e., a bell) with the outcome of salivation solely to the bell. Operant conditioning takes practices to combine conditioned stimuli (e.g., a keypress) with an unconditioned stimulus (e.g., food). In a series of trial and error, the animal learns to press the correct key to get the food award. Both learning procedures are accompanied with changings in the synaptic and neuronal connections in the brain, thus being defined as unconscious learning due to brain plasticity.

^18^It should be mentioned that this research has been subjected to critical examination in the last 2 years due to an evaluation by Reinhold Scherer, a BCI specialist of the University of Essex, U.K. He had found irregularities in the data analyses of the study and claimed: “Trials with locked-in patients are extremely expensive and logistically difficult, so it is hard for other groups to replicate the work. […] The hint that there might be a way to communicate with these patients is a welcome message, but there's just not enough evidence that we can definitely say it's working” (Vogel, 2019). These claims provoked a critical assessment of the studies of the Birbaumer group with completely locked-in patients subsidized by the German Funding Association (DFG) that led to the exclusion of Birbaumer and Chaudhadry from further funding due to scientific malpractice.

^19^https://blog.despatch.com/new-brain-computer-interface-tech-will-soon-let-unconscious-patients-communicate/ (accessed December 28, 2020).

^20^https://www.syfy.com/syfywire/unconscious-patients-can-now-sort-of-speak-to-us (accessed December 28, 2020).

^21^https://encyclopedia.pub/2963 (accessed January 3, 2021).

^22^http://stelarc.org/?catID=20265 (accessed December 28, 2020).

^23^http://news.bbc.co.uk/2/hi/health/4648139.stm (accessed December 28, 2020).

^24^https://www.youtube.com/watch?v=jA8inmHhx8c (accessed January 8, 2021).

^25^https://www.theguardian.com/science/2013/feb/28/brains-rats-connected-share-information (accessed April 25, 2017).

^26^In the heading “Telepathic rats team up across continents”, the Financial Times reported about the BTBI experiments, https://www.ft.com/content/422e4e8a-8197-11e2-904c-00144feabdc0 (accessed February 28, 2013).

^27^https://posthumanities.net/om/ (accessed January 6, 2021).
